# Initial Genome-Wide Characterization of Turkmen Individuals from the Dashoguz Region, Turkmenistan

**DOI:** 10.3390/biology15151244

**Published:** 2026-07-28

**Authors:** Zhassulan Zhaniyazov, Akmaral Kulatayeva, Aikorkem Mustafayeva, Bakytzhan Anapiyayev, Konirsha Iskakova, Utepbergen Bissenov, Aigerim Kassymbekova, Leyla Djansugurova

**Affiliations:** 1 Department of Chemical and Biochemical Engineering, Satbayev Kazakh National Research Technical University, Almaty 050013, Kazakhstan; 2 Institute of Genetics and Physiology, CS MSHE RK, Almaty 050060, Kazakhstanleylad@mail.ru (L.D.); 3 Department of Molecular Biology and Genetics, Al-Farabi Kazakh National University, Almaty 050040, Kazakhstan; 4 Faculty of Natural Sciences, Atyrau University, Atyrau 060011, Kazakhstan

**Keywords:** Turkmen, Central Asia, population genetics, genome-wide genotyping, genetic structure, runs of homozygosity, Y chromosome, mitochondrial DNA

## Abstract

Turkmen populations remain underrepresented in genome-wide reference datasets, limiting comparative analyses of genetic diversity in Central Asia. This study provides an initial regional genome-wide characterization of self-identified Turkmen students who were studying in Kazakhstan at the time of sample collection and who reported the Dashoguz Region, Turkmenistan, as their place of birth in the questionnaire. Approximately 650,000 markers were genotyped in 25 participants, of whom 23 unrelated individuals were retained after genotype quality control and relatedness filtering. Population structure was evaluated with 600 present-day Eurasian reference individuals using principal component analysis, ADMIXTURE v1.3.0, and pairwise Weir–Cockerham *F_ST_* estimates based on 17,471 linkage-disequilibrium-pruned autosomal SNPs. Runs of homozygosity, Y-chromosome haplogroups, and mitochondrial DNA haplogroups were also examined. The analyzed cohort was positioned within the Central Asian genetic landscape and showed the lowest pairwise differentiation from Uzbek, Tajik, and AADR Turkmen reference groups. Runs-of-homozygosity analysis showed mostly low-to-moderate individual burdens, although one individual had elevated long-segment homozygosity; sensitivity analyses indicated that the main autosomal results were not driven by this individual. Paternal and maternal haplogroup assignments revealed diverse uniparental lineages. These data provide a regional genome-wide resource for future comparative studies of Central Asian genetic diversity and have been deposited in the European Variation Archive.

## Simple Summary

Turkmen populations remain poorly represented in genome-wide genetic studies. In this study, we generated and analyzed genome-wide genotype data from 25 self-identified Turkmen students who were studying in Kazakhstan and who reported the Dashoguz Region, Turkmenistan, as their place of birth in the questionnaire. Of these, 23 unrelated individuals were retained after quality control. By comparing these individuals with present-day Eurasian reference populations, we describe their genetic position within the broader Central Asian context. The analyzed cohort was placed within the Central Asian genetic landscape and showed low genetic differentiation from neighboring Central Asian groups. Additional analyses of runs of homozygosity and paternal and maternal lineages revealed individual-level and lineage diversity within the cohort. The dataset has been deposited in the European Variation Archive and provides a regional genome-wide resource for future studies of Central Asian genetic diversity.

## 1. Introduction

Large-scale genomic projects have substantially expanded knowledge of human genetic variation and population history. Resources such as the 1000 Genomes Project, the Simons Genome Diversity Project, and high-coverage genome panels representing diverse human populations have become important frameworks for comparative population-genetic research [1,2,3]. Nevertheless, the geographical and population coverage of available genomic resources remains uneven, and many populations remain represented by few individuals or are absent from commonly used reference datasets [4,5].

Central Asia is particularly relevant in this context. Located between the Eurasian steppe, the Iranian plateau, Siberia, South Asia, the Caucasus, and the Near East, the region has long formed a zone of interaction among populations with diverse demographic, linguistic, and cultural histories. Genome-wide studies have demonstrated that Central Asian genetic diversity cannot be adequately described by a simple division between western and eastern Eurasia. Instead, present-day populations occupy overlapping genetic gradients shaped by long-term regional contacts, population movements, admixture, founder effects, and local continuity [6,7,8,9]. Ancient DNA studies have further shown that the demographic history of South and Central Asia involved multiple temporally distinct ancestry sources and episodes of migration and admixture [10,11].

Turkmen populations are of particular interest within this broader regional framework. Turkmenistan occupies a southwestern position in Central Asia, linking the Caspian region and the Iranian plateau with Afghanistan, Uzbekistan, and the inner Eurasian steppe. This geographical setting makes Turkmen populations informative for investigating genetic relationships across Central Asia and adjacent regions. At the same time, genome-wide data from Turkmen individuals originating from Turkmenistan remain limited, restricting direct assessment of their genetic affinities and diversity in comparison with neighboring Eurasian populations [4,5,12,13].

Previous genetic studies have provided valuable information on paternal and maternal lineage diversity in Central Asia [14]. A recent Y-chromosome study of Turkmen men from Turkmenistan reported high paternal haplotype diversity and genetic relationships with Turkmen groups sampled in neighboring regions [12]. However, Y-chromosome and mitochondrial DNA markers each represent only a single paternal or maternal line of descent and therefore cannot fully characterize genome-wide population structure or autosomal genetic relationships.

Genomic variation data are also available for Iranian Turkmen through the Iranome project, which used whole-exome sequencing to characterize coding variation in major ethnic groups residing in Iran [13]. These data provide an important resource for the Iranian Turkmen community but are not directly equivalent to dense genome-wide genotype data from individuals originating from Turkmenistan. The exome-focused design also provides a different marker framework from that required for analyses based on broadly distributed autosomal variants, such as principal component analysis, model-based clustering, pairwise genetic differentiation, and runs of homozygosity.

Genome-wide single-nucleotide polymorphism (SNP) data permit several complementary levels of analysis. Principal component analysis can characterize major axes of genetic variation and position individuals relative to reference populations [15]. Model-based clustering provides a comparative representation of genetic structure within a defined dataset [16], whereas pairwise fixation index (*F_ST_*) estimates quantify genetic differentiation between population groups [17]. Runs of homozygosity provide additional information on individual-level autozygosity and demographic processes [18,19]. Y-chromosome and mitochondrial DNA haplogroups can further complement autosomal analyses by describing paternal and maternal lineage diversity [12,14,20,21,22].

The aim of the present study was to provide an initial regional genome-wide characterization of self-identified Turkmen individuals who reported the Dashoguz Region, Turkmenistan, as their place of birth. We generated dense SNP-array genotype data, evaluated the position of the cohort within a present-day Eurasian reference panel using autosomal population-structure analyses, summarized individual-level runs of homozygosity, and inferred Y-chromosome and mitochondrial DNA haplogroups as complementary lineage markers. By depositing the resulting genotype data in the European Variation Archive, this study contributes a regional genome-wide resource for future comparative studies of genetic diversity in Central Asia.

## 2. Materials and Methods

### 2.1. Study Participants, Sample Collection, and Genotyping

The study included 25 self-identified Turkmen students who were studying in Kazakhstan at the time of sample collection and who reported the Dashoguz Region, Turkmenistan, as their place of birth in the questionnaire. The cohort therefore represents an available regional sample of Dashoguz-born Turkmen individuals rather than a population-based random sample of all Turkmen individuals from the Dashoguz Region or Turkmenistan as a whole. Peripheral venous blood samples were collected into EDTA-containing tubes, and written informed consent was obtained from all participants before sample collection.

Genomic DNA was extracted from peripheral blood using the Wizard Genomic DNA Purification Kit (Promega, Madison, WI, USA) according to the manufacturer’s instructions. DNA concentration and purity were assessed using a NanoDrop One spectrophotometer (Thermo Fisher Scientific, Waltham, MA, USA). Genotyping was performed using the Illumina Global Screening Array v3 on an Illumina iScan platform (Illumina, San Diego, CA, USA). Genotype calling was carried out in GenomeStudio 2.0, and the resulting data were exported in PLINK binary format. Variant coordinates were based on the GRCh37/hg19 human reference genome assembly.

### 2.2. Genotype Quality Control

Genotype quality control was performed using PLINK v1.9 [23]. The analysis was limited to autosomal SNPs. Individuals and variants with genotype missingness exceeding 2% were excluded from further analysis.

One of the 25 initially genotyped individuals did not meet the sample-level quality threshold and was removed. After missingness filtering, the dataset comprised 24 individuals and 593,197 SNPs, with an overall genotyping rate of 99.22%.

Individual heterozygosity was examined to identify possible outliers, and no additional samples were excluded at this stage. Genetic relatedness was assessed using identity-by-descent estimates derived from a linkage-disequilibrium-pruned set of SNPs. One individual from a closely related pair was subsequently removed. The final quality-controlled dataset consisted of 23 unrelated Turkmen individuals and was used in the downstream population-genetic analyses.

### 2.3. Reference Population Panel and Genotype Harmonization

A reference panel of 600 present-day individuals was selected from the Human Origins dataset available through the Allen Ancient DNA Resource (AADR) [24]. The panel included populations from Europe, the Caucasus, the Middle East, South Asia, Central Asia, Northeast Eurasia, and East Asia, providing a broad comparative framework for assessing the genetic position of the study participants. The AADR panel included seven Turkmen individuals sampled in Uzbekistan, who were treated as an external reference group distinct from the Dashoguz Turkmen individuals analyzed in the present study. Reference-population sample sizes are provided in Supplementary Table S1, and the full composition of the selected Human Origins/AADR reference panel is provided in Supplementary Table S2.

The Turkmen and reference datasets were harmonized using shared autosomal biallelic SNPs. Variants were matched by genomic position and allele correspondence. Duplicated, multiallelic, strand-ambiguous, and otherwise incompatible variants were excluded to minimize potential errors arising from differences between genotyping platforms.

Following harmonization and variant-level missingness filtering, 24,086 autosomal SNPs shared between the Turkmen and Human Origins datasets were retained. A regional analytical panel was then formed by combining the 23 Turkmen individuals with 600 selected reference individuals. Variants with a minor allele frequency below 5% were excluded, followed by linkage disequilibrium pruning using a window of 200 SNPs, a step size of 25 SNPs, and an r^2^ threshold of 0.2. The final dataset comprised 623 individuals and 17,471 autosomal SNPs and was used for principal component analysis, ADMIXTURE, and pairwise *F_ST_* estimation.

### 2.4. Principal Component Analysis

Principal component analysis was used to characterize the major axes of genetic variation and to position the study participants within the broader genetic diversity of Eurasian populations. The analysis was performed jointly for the 23 Dashoguz Turkmen individuals and 600 reference individuals using 17,471 linkage-disequilibrium-pruned autosomal SNPs. Principal components were estimated in PLINK v1.9 [23], following established applications of principal component analysis to genome-wide population structure [15].

The first two principal components were used to visualize population structure. The proportion of genetic variation explained by each component was derived from the corresponding eigenvalues. Turkmen individuals from the present study and Turkmen samples available in the AADR reference dataset were displayed separately to allow direct comparison.

### 2.5. Model-Based Clustering Analysis

Model-based clustering was performed with ADMIXTURE v1.3.0 [16] using the LD-pruned regional dataset described above. Models with K values ranging from 2 to 10 were evaluated using 10-fold cross-validation. The solutions with the lowest cross-validation errors were examined across repeated runs with different random seeds, and the run with the lowest cross-validation error was used for visualization.

Individuals were grouped by population and broad geographic region. Within each population, samples were ordered according to their inferred component proportions. Turkmen individuals from the present study and Turkmen reference individuals from AADR were displayed as separate groups.

The inferred components were interpreted as model-dependent summaries of genetic structure within the analyzed dataset and not as direct estimates of ancestry from discrete contemporary or historical populations.

ADMIXTURE cross-validation results for K = 2–10 are provided in Supplementary Table S3, and mean component proportions by population at K = 4 are summarized in Supplementary Table S4.

### 2.6. Pairwise Genetic Differentiation

Pairwise genetic differentiation between the study Turkmen cohort and each reference population was estimated using the Weir–Cockerham *F_ST_* estimator [17] implemented in PLINK v1.9 [23]. The analysis was performed using the LD-pruned regional dataset described above.

For each comparison, the 23 Turkmen individuals retained after quality control were analyzed jointly with a single reference population. Weighted *F_ST_* estimates were used for population-level comparisons.

As a sensitivity analysis, principal component analysis and pairwise *F_ST_* estimation were repeated after excluding the individual with the highest runs-of-homozygosity burden. This analysis was performed to assess whether the main autosomal population-structure results were influenced by a a single individual with a high runs-of-homozygosity (ROH) burden. The same reference panel, marker set, and analytical procedures were used as in the primary principal component analysis (PCA) and *F_ST_* analyses.

### 2.7. Runs of Homozygosity Analysis

Runs of homozygosity were identified in the 23 Turkmen individuals using PLINK v1.9, following established genome-wide approaches to ROH detection [18,19]. ROH calling was performed on the autosomal genotype dataset after quality control and before linkage disequilibrium pruning.

A homozygous segment was required to contain at least 100 SNPs and to span a minimum length of 1 Mb. The maximum gap between consecutive SNPs was set to 1000 kb, and a minimum density of one SNP per 50 kb was required. One heterozygous genotype and up to five missing genotypes were permitted within the sliding window.

For each individual, the number of ROH segments, cumulative ROH length, mean segment length, and maximum segment length were calculated. The genomic inbreeding coefficient based on runs of homozygosity (F_ROH_) was calculated as the cumulative ROH length divided by an autosomal genome length of 2769 Mb. ROH segments were grouped into five length categories: 1–2 Mb, 2–4 Mb, 4–8 Mb, 8–16 Mb, and >16 Mb. Individual-level runs-of-homozygosity summary statistics are provided in Supplementary Table S5.

For supplementary annotation, genomic coordinates of all ROH segments were intersected with gene annotations from GENCODE release 19 [25], corresponding to the GRCh37/hg19 reference assembly. For each ROH segment, overlapping genes and protein-coding genes were recorded. Recurrent ROH regions were defined by merging overlapping ROH segments across individuals on the same chromosome, and cohort frequency was calculated as the number of individuals carrying an ROH in the merged region divided by the 23 individuals retained after quality control.

### 2.8. Y-Chromosome Haplogroup Assignment

Y-chromosome haplogroups were inferred using Yhaplo v2.1.18 [26]. The analysis was restricted to male individuals retained after genotype quality control and relatedness filtering. The final Y-chromosome analysis included 19 male individuals.

For each male participant, the haplogroup associated with the representative phylogenetic SNP reported by Yhaplo was retained as the final assignment. Derived- and ancestral-SNP reports were reviewed to assess the consistency of the inferred lineages. Haplogroup counts were summarized descriptively. Individual-level Y-chromosome haplogroup assignments are provided in Supplementary Table S6.

### 2.9. Mitochondrial DNA Haplogroup Assignment

Mitochondrial markers were extracted from the original SNP-array dataset. After harmonization against the revised Cambridge Reference Sequence (rCRS; NC_012920.1) [20], duplicate probes, non-standard allele records, and discordant calls were removed, leaving 985 unique mitochondrial positions for haplogroup inference.

A sample-specific HSD file was generated from the observed variants and the positions assessed by the genotyping array. Mitochondrial haplogroups were assigned using HaploGrep 3.3.2 [21] with the PhyloTree 17 Forensic Update rCRS-based tree, version 1.2, and the Kulczynski distance measure [22]. Detailed subclade assignments and HaploGrep quality scores are provided in Supplementary Table S7, whereas major mitochondrial haplogroups are summarized in the main text. A genotype quality-control summary of the initially genotyped Turkmen samples is provided in Supplementary Table S8.

## 3. Results

### 3.1. Principal Component Analysis

Principal component analysis positioned the study participants within the broader Eurasian genetic landscape. PC1 primarily reflected a west-to-east Eurasian gradient, separating European and East Asian populations, while populations from the Caucasus, the Middle East, South Asia, Central Asia, and Northeast Eurasia occupied intermediate positions.

**Figure 1:**
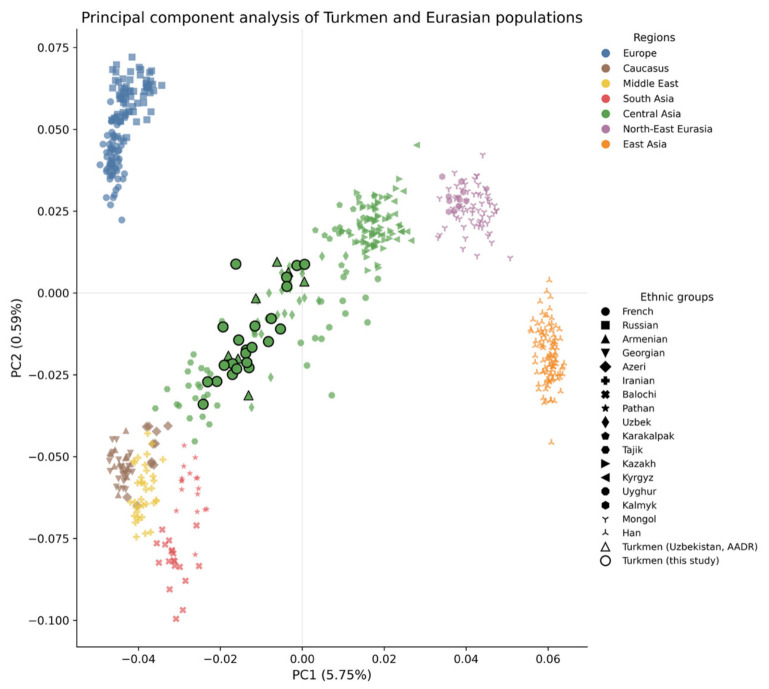
Principal component analysis of Turkmen individuals and modern Eurasian reference populations. The analysis included 23 Dashoguz Turkmen individuals from the present study and 600 modern reference individuals from the Allen Ancient DNA Resource. Colors indicate broad geographic regions, while marker shapes distinguish individual populations. Turkmen individuals from the present study are shown as large highlighted circles, and AADR Turkmen samples are shown as triangles.

The 23 Dashoguz Turkmen individuals retained after quality control were distributed within the Central Asian genetic space and followed the broader Central Asian cline. Their positions overlapped partly with the AADR Turkmen reference samples and with several neighboring Central Asian populations (Figure 1). PC1 and PC2 explained 5.75% and 0.59% of the total genetic variation, respectively.

As a sensitivity analysis, PCA was repeated after excluding the individual with the highest runs-of-homozygosity burden. The overall placement of the remaining Turkmen individuals within the Central Asian genetic space remained unchanged, indicating that the main PCA pattern was not driven by this individual (Supplementary Figure S1).

### 3.2. Model-Based Clustering Analysis

**Figure 2:**
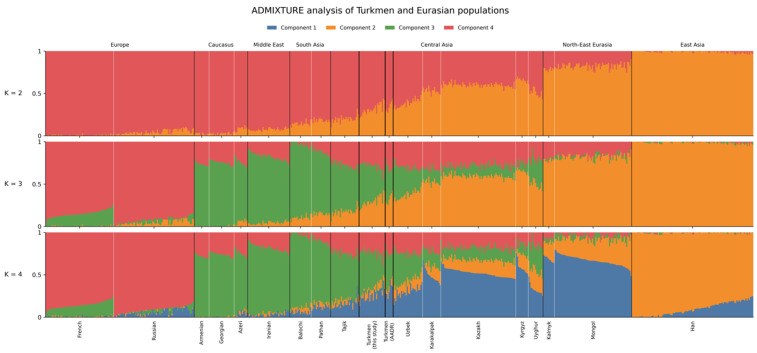
ADMIXTURE analysis of Turkmen individuals and modern Eurasian reference populations at K = 2–4. Each vertical bar represents one individual, and colored segments indicate the proportions assigned to model-based components. Individuals are grouped by population and broad geographic region. Dashoguz Turkmen individuals from the present study and AADR Turkmen samples are highlighted by vertical boundary lines. K = 4 produced the lowest cross-validation error among the tested models and was selected for presentation. Component colors represent statistical clusters inferred from the analyzed dataset and should not be interpreted as direct ancestry proportions from specific contemporary or historical populations.

ADMIXTURE analysis revealed progressively finer-scale patterns of population structure across K = 2–4 (Figure 2). At K = 2, the inferred component proportions broadly followed the west-to-east Eurasian gradient observed in the principal component analysis. At K = 3, an additional component was most prominent among populations from the Caucasus, the Middle East, and South Asia. At K = 4, a further component was enriched among Northeast Eurasian and several Central Asian populations.

Among the tested models from K = 2 to K = 10, K = 4 yielded the lowest cross-validation error and was selected for presentation. At K = 4, the Dashoguz Turkmen individuals displayed a mixed component profile. The largest mean proportion corresponded to Component 3 (41.84%), followed by Component 1 (26.63%), Component 4 (24.15%), and Component 2 (7.37%). The AADR Turkmen reference samples showed a broadly similar profile, with mean proportions of 38.38%, 31.80%, 23.51%, and 6.31% for Components 3, 1, 4, and 2, respectively.

The component profile of the Dashoguz Turkmen cohort was also broadly comparable to that of the Uzbek reference group, although differences in mean component proportions were observed. Tajik individuals showed a higher mean proportion of Component 3, whereas Kazakh, Kyrgyz, and Karakalpak individuals showed higher proportions of Component 1. Uyghur individuals displayed a comparatively higher proportion of Component 2.

These components represent statistical clusters inferred from the analyzed reference panel and should not be interpreted as direct estimates of ancestry from specific contemporary or historical populations.

### 3.3. Pairwise Genetic Differentiation

**Figure 3:**
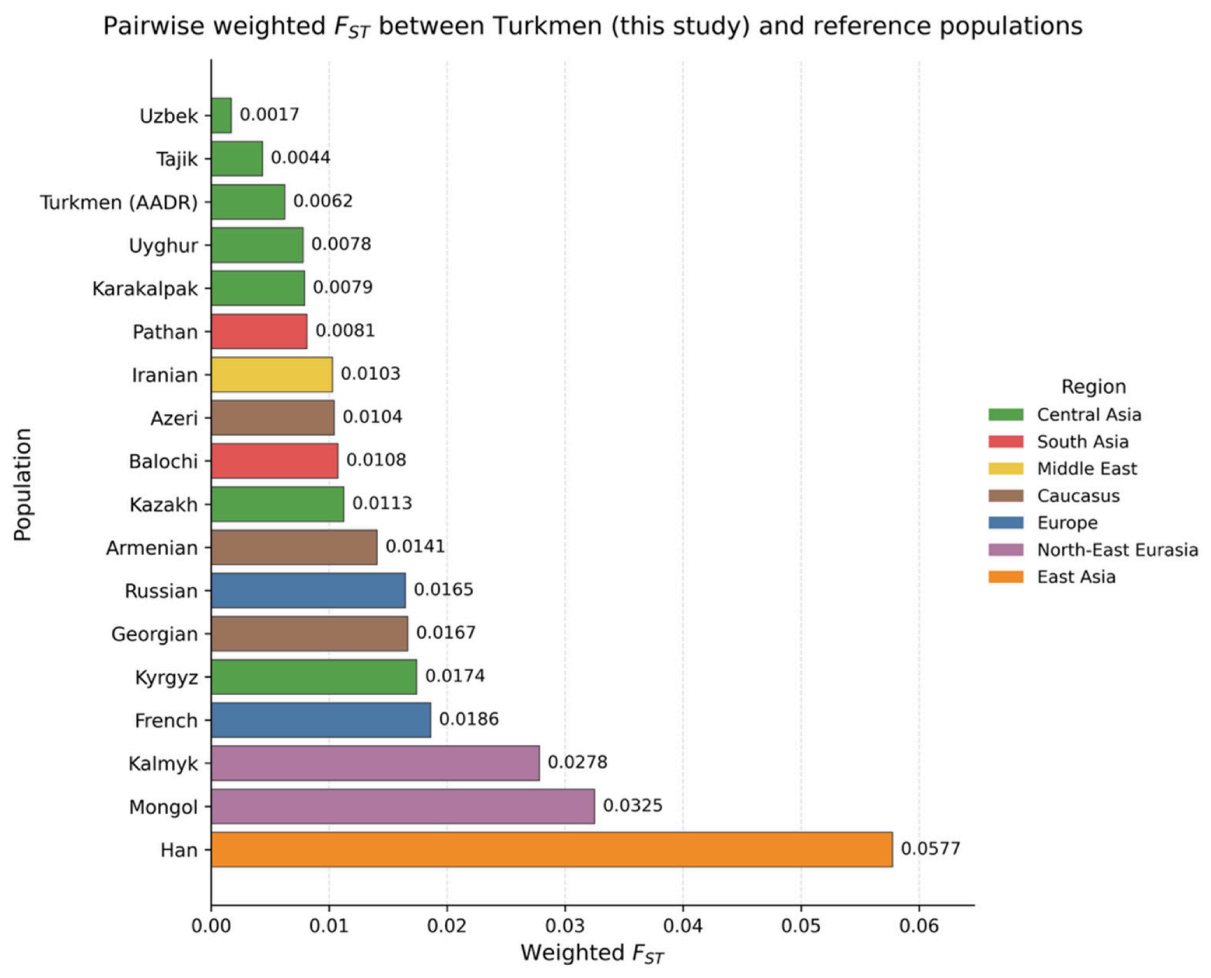
Pairwise weighted *F_ST_* estimates between the Dashoguz Turkmen cohort and modern Eurasian reference populations. Bars are ordered from the lowest to the highest genetic differentiation. Colors indicate the broad geographic region assigned to each reference population. Weighted *F_ST_* values were calculated using the Weir–Cockerham estimator on the linkage-disequilibrium-pruned dataset.

Pairwise weighted *F_ST_* estimates indicated low differentiation between the Dashoguz Turkmen cohort and several neighboring Central Asian reference populations (Figure 3). The lowest weighted *F_ST_* was observed with the Uzbek reference population (*F_ST_* = 0.0017), followed by Tajik (*F_ST_* = 0.0044), AADR Turkmen (*F_ST_* = 0.0062), Uyghur (*F_ST_* = 0.0078), and Karakalpak (*F_ST_* = 0.0079).

Intermediate differentiation was observed with Pathan, Iranian, Azeri, Balochi, and Kazakh populations, with weighted *F_ST_* values ranging from 0.0081 to 0.0113. Higher estimates were observed for Armenian (*F_ST_* = 0.0141), Russian (*F_ST_* = 0.0165), Georgian (*F_ST_* = 0.0167), Kyrgyz (*F_ST_* = 0.0174), and French (*F_ST_* = 0.0186) reference populations.

The highest differentiation was observed with Kalmyk (*F_ST_* = 0.0278), Mongol (*F_ST_* = 0.0325), and Han (*F_ST_* = 0.0577) populations. Overall, the *F_ST_* results were consistent with the placement of the Dashoguz Turkmen cohort within the Central Asian genetic landscape, with the highest differentiation observed for the Northeast Eurasian and East Asian reference groups included in the analysis.

The estimate obtained for the AADR Turkmen group should be interpreted cautiously because this reference group contained only seven individuals. In the sensitivity analysis excluding the individual with the highest runs-of-homozygosity burden, the ordering of the closest reference populations remained unchanged, and the differences from the full-cohort *F_ST_* estimates were minimal (Supplementary Table S9).

### 3.4. Runs of Homozygosity

**Figure 4:**
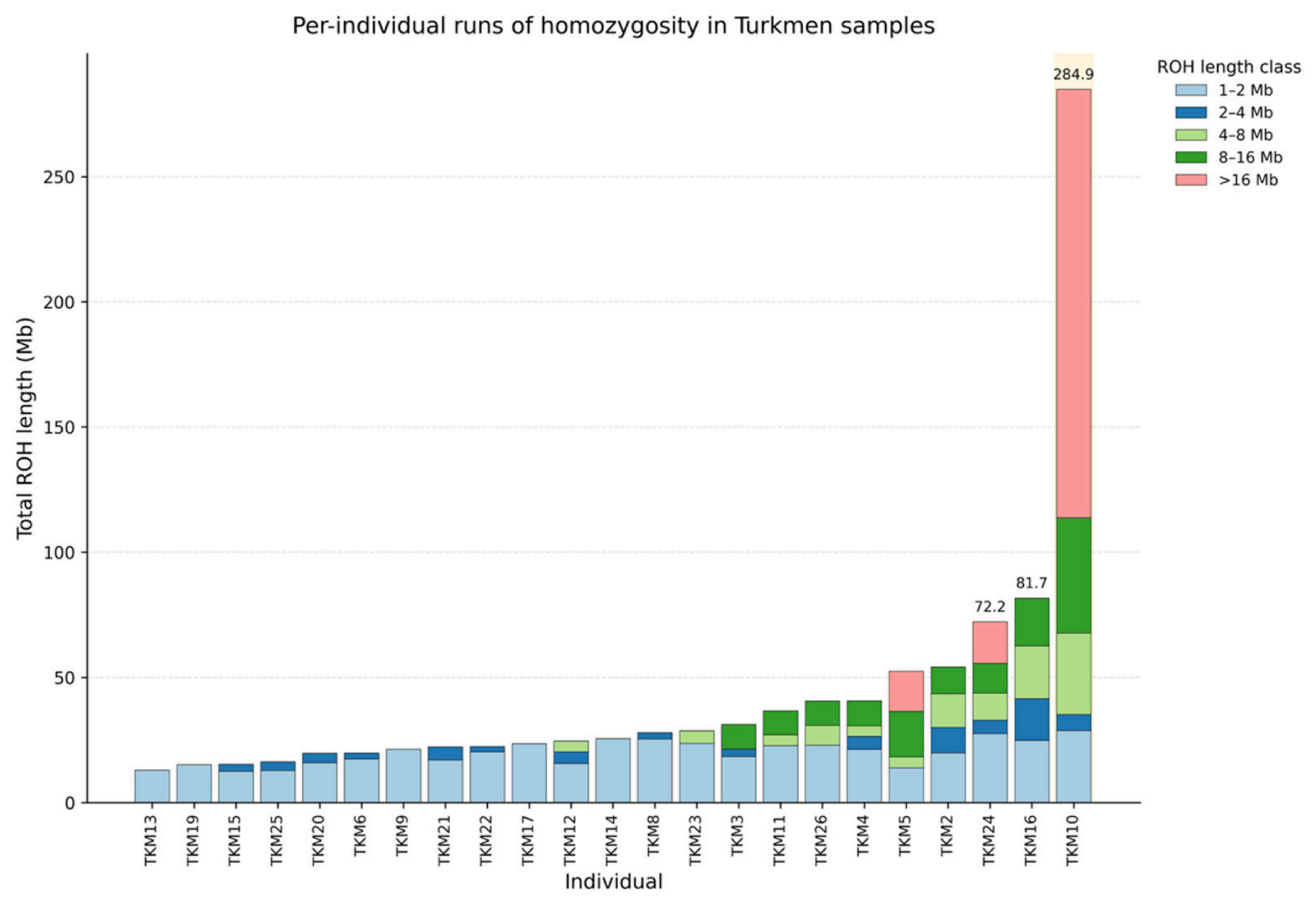
Per-individual runs of homozygosity in the Turkmen study samples. Stacked bars show the cumulative ROH length for each individual, partitioned into five segment-length categories: 1–2 Mb, 2–4 Mb, 4–8 Mb, 8–16 Mb, and >16 Mb. Individuals are ordered by increasing cumulative ROH length. One individual exhibited a markedly elevated ROH burden, including several segments longer than 16 Mb. The yellow-highlighted value indicates the total cumulative ROH length of the individual with the highest ROH burden.

A total of 429 runs of homozygosity were identified among the 23 Turkmen individuals (Figure 4). The median number of ROH segments per individual was 18.0, with an interquartile range of 15.0–20.5. The median cumulative ROH length was 25.53 Mb, with an interquartile range of 20.53–40.60 Mb. The median F_ROH_ was 0.00922, with an interquartile range of 0.00741–0.01466. For most individuals, the ROH burden consisted predominantly of short segments between 1 and 2 Mb. Three individuals carried one or more ROH segments longer than 16 Mb.

One individual showed a markedly elevated ROH burden, with 40 segments spanning a cumulative length of 284.90 Mb and an F_ROH_ of 0.10285. The longest segment in this individual measured 41.12 Mb, while five segments longer than 16 Mb accounted for 171.14 Mb of the total ROH length. Two other individuals showed the next highest cumulative ROH lengths, at 81.68 Mb and 72.22 Mb, respectively.

Overall, the ROH profiles of most participants were dominated by shorter segments, whereas one sample showed a distinct excess of long ROH segments and a substantially higher genome-wide ROH burden than the remaining individuals. This pattern is consistent with recent autozygosity at the individual level and should not be generalized to the wider Turkmen population.

Genomic coordinates, length categories, and overlapping gene content for all ROH segments are provided in Supplementary Table S10, while recurrent ROH regions observed in at least two individuals, their cohort frequencies, and overlapping gene content are summarized in Supplementary Table S11.

### 3.5. Y-Chromosome and Mitochondrial DNA Haplogroup Diversity

Y-chromosome haplogroups were assigned to the 19 male individuals retained in the final dataset. At the major haplogroup level, haplogroup R was observed in seven individuals, followed by N in five individuals and J in four individuals. Haplogroups C, L, and T were each represented by one individual. More detailed classification identified 12 paternal lineages, with R-L266 and N-L735 being the most frequently observed branches. The remaining paternal lineages were represented by one or two individuals each.

Mitochondrial DNA haplogroups were assigned to all 23 individuals retained in the final dataset. HaploGrep quality scores ranged from 0.9063 to 1.0000, with a mean score of 0.9788, and no sample-level warnings were reported. At the major haplogroup level, haplogroup H was observed in five individuals, haplogroups J and U in three individuals each, and haplogroups D, M, and T in two individuals each. Haplogroups A, B, C, F, G, and I were each observed in one individual.

Overall, the paternal and maternal lineage analyses indicated diverse uniparental haplogroup composition within the analyzed Dashoguz Turkmen cohort. Given the sample size, these results are presented as descriptive lineage summaries rather than as population-wide haplogroup frequency estimates.

## 4. Discussion

This study provides an initial regional genome-wide characterization of Turkmen individuals from the Dashoguz Region, Turkmenistan. Principal component analysis, model-based clustering, and pairwise genetic differentiation consistently placed the analyzed cohort within the broader Central Asian genetic landscape described in previous genome-wide studies [6,7,8,9]. Within the reference panel used here, the lowest differentiation was observed with Uzbek, Tajik, and AADR Turkmen groups. At the same time, the ROH and uniparental lineage analyses revealed considerable variation among individuals within the cohort.

The concordance among PCA, ADMIXTURE, and *F_ST_* strengthens the overall interpretation of the autosomal results. In the PCA, the Dashoguz Turkmen individuals occupied an intermediate position along the broader west-to-east Eurasian gradient and overlapped partly with other Central Asian populations rather than forming a clearly isolated cluster. This pattern is consistent with previous studies showing that Central Asian genetic diversity reflects long-term population contact, repeated migrations, and gradual transitions between western and eastern Eurasian genetic variation [6,7,8,9]. Genetic relationships in this region therefore frequently extend across present-day national, linguistic, and ethnic boundaries [6,7,8,9,14].

Pairwise *F_ST_* estimates supported the genetic placement of the Dashoguz Turkmen cohort within the broader Central Asian landscape. The lowest differentiation was observed with the Uzbek reference population, followed by the Tajik and AADR Turkmen groups. This pattern is consistent with geographical proximity and long-standing demographic interactions among populations of Central Asia and adjacent regions. Previous studies have likewise reported extensive gene flow among neighboring populations, together with substantial genetic heterogeneity both among and within broadly defined Central Asian and Turkic-speaking groups [6,7,8,14,27].

The ordering of the lowest *F_ST_* estimates should be interpreted in the context of the reference panel used in this study. In particular, the lower estimate for the Uzbek group than for the AADR Turkmen group does not imply that the analyzed cohort is generally more closely related to Uzbeks than to other Turkmen populations. The AADR Turkmen reference group comprised only seven individuals sampled in Uzbekistan and may therefore not capture the broader genetic diversity of Turkmen populations. Its *F_ST_* estimate should consequently be interpreted cautiously because of the small reference sample and possible local sampling effects. The sensitivity analysis excluding the individual with the highest ROH burden produced the same ordering of the closest reference populations, indicating that the main pairwise differentiation results were not driven by this individual.

The ADMIXTURE analysis provided a complementary model-based representation of population structure. At K = 4, which yielded the lowest cross-validation error among the tested models, the component proportions observed in the Dashoguz Turkmen cohort were broadly similar to those of the AADR Turkmen and Uzbek reference groups. This pattern is consistent with previous evidence that Central Asian and Turkic-speaking populations have complex demographic histories shaped by repeated population interactions and gene flow [6,8].

The inferred ADMIXTURE components should not be assigned direct ethnic, geographical, or historical identities [28]. Their proportions depend on the composition of the reference panel, the marker set, data-processing procedures, and the selected value of K. They are therefore most informative for comparisons within the present analytical framework and do not independently identify particular ancient populations or migration events. Formal reconstruction of demographic sources and admixture history would require broader sampling and dedicated analyses incorporating appropriate ancient DNA reference populations from Central Asia, the Eurasian steppe, and adjacent regions [10,11].

The ROH analysis revealed marked inter-individual variation that was not apparent from the population-level structure analyses. In most participants, the ROH burden was dominated by short segments, whereas a small number of individuals carried longer segments and higher cumulative ROH lengths. Short ROH are generally associated with more distant shared ancestry and long-term demographic processes, whereas long ROH more often reflect recent parental relatedness [18,19,29].

One individual showed the highest ROH burden in the cohort, with an elevated F_ROH_ value and multiple long ROH segments. This pattern is consistent with a comparatively high level of recent autozygosity. Because it was observed in a single individual, it should not be generalized to the wider Turkmen population. Two other individuals showed the next highest cumulative ROH lengths, although their values were substantially lower than that observed in the high-ROH individual.

Absolute ROH estimates are influenced by marker density, genomic coverage, genotype quality, and the parameters used to define homozygous segments [18,19,29]. Comparisons with previously studied populations should therefore be made cautiously and, where possible, restricted to datasets generated and analyzed using comparable platforms and ROH-calling criteria. In the present study, the ROH results are most informative for assessing variation among individuals within the same dataset.

The lineage-specific analyses provided additional evidence of paternal and maternal heterogeneity within the cohort. At the major Y-chromosome haplogroup level, haplogroups R, N, and J were the most frequently observed groups among the 19 male participants, while haplogroups C, L, and T were each represented by one individual. More detailed classification identified 12 paternal lineages, with R-L266 and N-L735 being the most frequently observed branches. This pattern is consistent with previous evidence of substantial paternal-lineage diversity among Turkmen men, although direct comparison with earlier studies is limited by differences in sample size, regional representation, marker systems, and phylogenetic resolution [12].

Mitochondrial DNA analysis similarly indicated maternal-lineage diversity within the cohort. Haplogroup H was the most frequently observed major maternal group, followed by J and U, while D, M, T, A, B, C, F, G, and I were also represented. Detailed classification identified 22 distinct mitochondrial subclade assignments among the 23 participants, with all but one represented by a single individual. Given the modest sample size, the observed Y-chromosome and mitochondrial DNA counts should be interpreted as descriptive characteristics of the analyzed cohort rather than as population-wide haplogroup frequency estimates.

Y-chromosome and mitochondrial DNA markers complement autosomal analyses by describing single paternal and maternal lines of descent [12,14,20,21,22]. However, they do not represent an individual’s overall genomic ancestry and should not be used as substitutes for genome-wide autosomal analyses.

An important strength of this study is the generation of dense genome-wide genotype data for Turkmen individuals from the Dashoguz Region, a regional group that remains poorly represented in international genomic resources [4,5,12,13]. The combined analysis of autosomal population structure, pairwise genetic differentiation, runs of homozygosity, and paternal and maternal haplogroups provided complementary information on both population-level affinities and individual-level diversity. The concordant PCA, ADMIXTURE, and *F_ST_* results support the placement of this regional Turkmen cohort within the broader Central Asian genetic landscape, whereas the ROH and uniparental marker analyses highlight variation among individuals and lineages within the study sample. The deposition of the genotype data in the European Variation Archive further increases the value of the dataset as a resource for future comparative studies.

Several limitations should be considered. First, only 23 participants remained after quality control, including 19 males available for Y-chromosome analysis. In addition, the participants were not recruited through a population-based sampling design. All participants were self-identified Turkmen students who were studying in Kazakhstan at the time of sample collection and who reported the Dashoguz Region, Turkmenistan, as their place of birth in the questionnaire. Information on parental and grandparental birthplace, tribal or clan affiliation, rural/urban origin, language use, and detailed community background was not systematically collected. These variables were therefore not used for stratification. Accordingly, the findings should be interpreted as an initial regional genome-wide characterization of the analyzed Dashoguz-born Turkmen cohort rather than as population-wide estimates for all Turkmen groups from Turkmenistan. The sample size and regional focus also limit the precision of haplogroup-count summaries and prevent evaluation of broader geographical, tribal, or community-level substructure.

Second, harmonization with the Human Origins reference panel reduced the dataset to 24,086 shared autosomal variants before regional population selection and LD pruning. Differences in marker ascertainment between genotyping platforms, together with the restricted set of overlapping variants, may influence PCA, ADMIXTURE, and *F_ST_* results. The reported patterns should therefore be interpreted within the context of the reference populations and marker set used in this study. This marker set is suitable for broad population positioning but limits fine-scale inference and formal demographic modeling. Finally, Y-chromosome and mitochondrial DNA haplogroups were inferred from SNP-array markers rather than complete sequence data. Although the available variants supported informative lineage assignments, genotyping arrays do not capture all downstream phylogenetic markers, Y-chromosome structural variation, or mitochondrial heteroplasmy. Some terminal assignments may therefore be refined by high-coverage sequencing.

Overall, this study expands the limited genome-wide information available for Turkmen individuals from the Dashoguz Region, Turkmenistan. The autosomal analyses consistently placed the analyzed cohort within the broader Central Asian genetic landscape, while the ROH and uniparental lineage results revealed considerable variation among individuals within the study sample. Broader and more geographically balanced sampling, combined with sequence-based data and ancient DNA comparisons, will be important for refining these findings and investigating Turkmen population history at higher resolution.

## 5. Conclusions

This study provides an initial regional genome-wide characterization of self-identified Turkmen individuals who reported Dashoguz Region, Turkmenistan, as their place of birth. Principal component analysis, ADMIXTURE, and pairwise genetic differentiation consistently positioned the analyzed cohort within the broader Central Asian genetic landscape, with the lowest differentiation observed relative to Uzbek, Tajik, and AADR Turkmen reference groups within the reference panel used here. Sensitivity analyses excluding the individual with the highest runs-of-homozygosity burden produced the same broad interpretation of the autosomal results, supporting the robustness of the main population-structure findings within this dataset.

Runs-of-homozygosity analysis and Y-chromosome and mitochondrial DNA haplogroup assignments revealed individual-level and uniparental lineage variation within the cohort. The findings should be interpreted as a regional genomic characterization of the analyzed Dashoguz-born Turkmen individuals rather than as population-wide estimates for all Turkmen groups from Turkmenistan. By depositing the genotype data in the European Variation Archive, this study provides a resource for future comparative analyses of Central Asian genetic diversity. Broader geographic sampling, sequence-based data, and integration with relevant ancient DNA datasets will help refine the genetic characterization of Turkmen populations and their demographic history.

## Data Availability

The genotype data generated in this study have been deposited in the European Variation Archive (EVA) at EMBL-EBI [30] under project accession number PRJEB115316 and will be made available in accordance with the repository release policy. Processed summary data supporting the reported results are provided in the Supplementary Materials. The Human Origins reference data used for comparative population-genetic analyses were obtained from the Allen Ancient DNA Resource.

## Supplementary Materials

The following supporting information can be downloaded at: https://www.mdpi.com/article/10.3390/biology15151244/s1, Figure S1: Principal component analysis after exclusion of the individual with the highest runs-of-homozygosity burden; Table S1: Reference-population sample sizes and pairwise weighted *F_ST_* estimates between the Dashoguz Turkmen cohort and Eurasian reference populations; Table S2: Composition of the selected Human Origins/AADR reference panel used for PCA, ADMIXTURE, and *F_ST_* analyses; Table S3: ADMIXTURE cross-validation results for K = 2–10; Table S4: Mean ADMIXTURE component proportions by population at K = 4; Table S5: Individual-level runs-of-homozygosity summary for the Turkmen individuals retained after quality control; Table S6: Individual Y-chromosome haplogroup assignments for male Turkmen individuals retained after quality control; Table S7: Individual mitochondrial DNA haplogroup assignments and HaploGrep quality scores for Turkmen individuals retained after quality control; Table S8: Genotype quality-control summary of the initially genotyped Turkmen samples; Table S9: Sensitivity analysis of pairwise weighted *F_ST_* estimates after exclusion of the individual with the highest runs-of-homozygosity burden; Table S10: Genomic coordinates, length categories, and overlapping gene content of all runs of homozygosity detected in the Turkmen individuals retained after quality control; Table S11: Recurrent runs-of-homozygosity regions observed in at least two individuals, their cohort frequency, and overlapping gene content.

## Abbreviations

The following abbreviations are used in this manuscript:

AADR	Allen Ancient DNA Resource
EVA	European Variation Archive
F_ROH_	Genomic inbreeding coefficient based on runs of homozygosity
*F_ST_*	Fixation index
GRCh37	Genome Reference Consortium Human Build 37
IBD	Identity by descent
LD	Linkage disequilibrium
mtDNA	Mitochondrial DNA
PCA	Principal component analysis
QC	Quality control
ROH	Runs of homozygosity
SNP	Single-nucleotide polymorphism
