# Supplementary material for: Initial Genome-Wide Characterization of Turkmen Individuals from the Dashoguz Region, Turkmenistan
Source: Biology (Basel). 2026 Jul 28;15(15):1244. doi: 10.3390/biology15151244 (PMC13465274; doi:10.3390/biology15151244)
Supplement: Supplementary file 1 [file biology-15-01244-s001.zip › Figure S1.pdf]

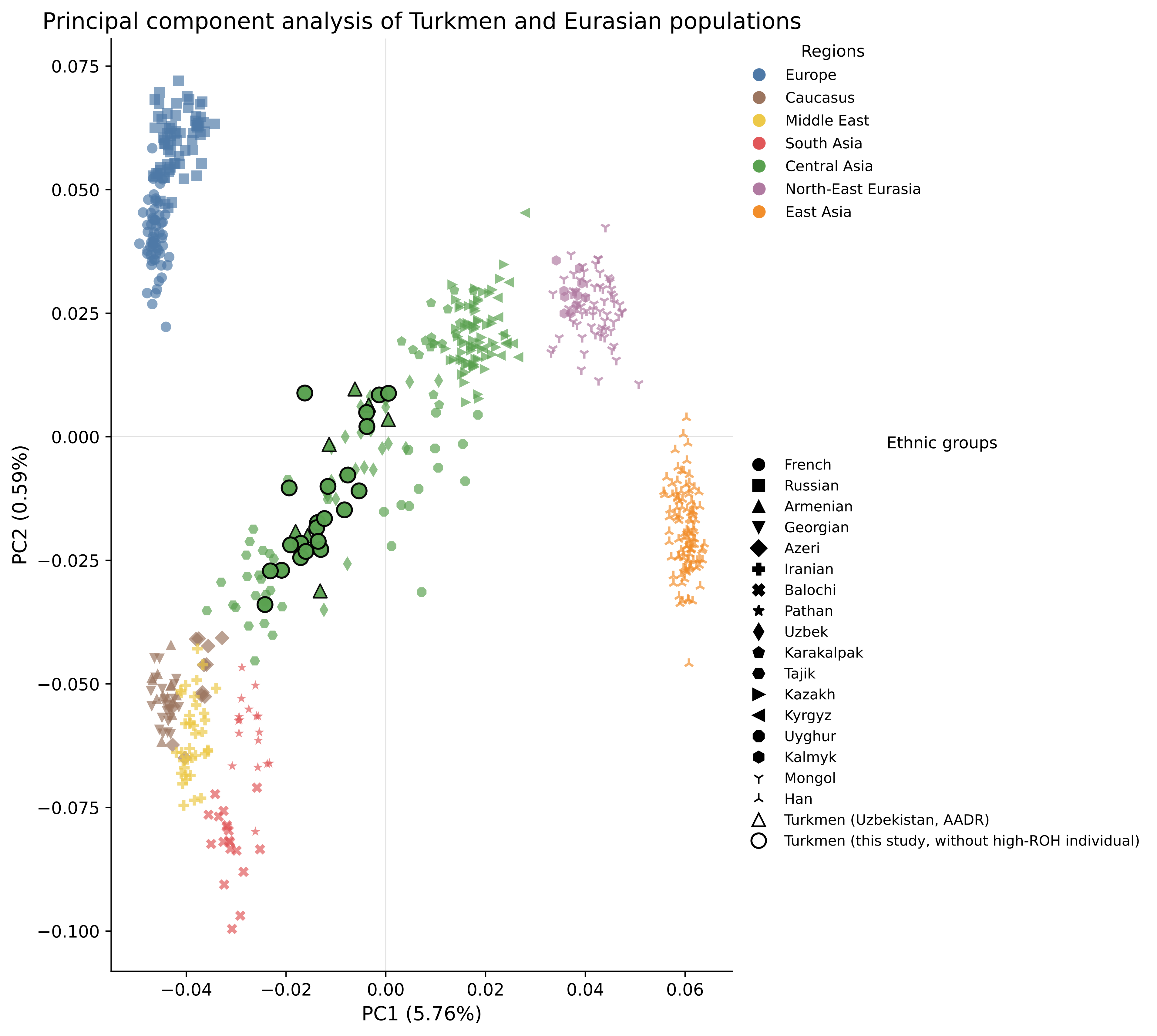
Principal component analysis of Turkmen and Eurasian populations
PC1 (5.76%)
PC2 (0.59%)
0.075
0.050
0.025
0.000
−0.025
−0.050
−0.075
−0.100
−0.04
−0.02
0.00
0.02
0.04
0.06
Regions
Europe
Caucasus
Middle East
South Asia
Central Asia
North-East Eurasia
East Asia
Ethnic groups
French
Russian
Armenian
Georgian
Azeri
Iranian
Balochi
Pathan
Uzbek
Karakalpak
Tajik
Kazakh
Kyrgyz
Uyghur
Kalmyk
Mongol
Han
Turkmen (Uzbekistan, AADR)
Turkmen (this study, without high-ROH individual)
